# Age patterns of Kaposi's sarcoma incidence in a cohort of HIV-infected men

**DOI:** 10.1002/cam4.312

**Published:** 2014-08-20

**Authors:** Hung N Luu, E Susan Amirian, Elizabeth Y Chiao, Michael E Scheurer

**Affiliations:** 1 Disivion of Epidemiology, Department of Medicine, School of Medicine, Vanderbilt UniversityNashville, Tennessee; 2 Dan L. Duncan Cancer Center, Baylor College of MedicineHouston, Texas; 3 Department of Pediatrics, Baylor College of MedicineHouston, Texas; 4 Department of Medicine, Baylor College of MedicineHouston, Texas; 5 Houston Health Services Research and Development Center of Excellence, Michael E. DeBakey Veterans Affairs Medical CenterHouston, Texas

**Keywords:** HIV-infected men, incidence patterns by age, Kaposi's sarcoma

## Abstract

The life expectancy for HIV-positive individuals has improved over time due to increasing access to highly active antiretroviral therapy (HAART). Yet, as the HIV-positive population ages, their risk of developing cancers also increases. Studies of Kaposi's sarcoma (KS) among elderly HIV-infected persons are quite limited. We examined the age patterns of KS incidence and an association between age and KS risk in a US cohort of 3458 HIV-infected men, the Multicenter AIDS Cohort Study (MACS). Poisson distribution was used to calculate incidence rates and respective 95% confidence intervals (95% CIs). Cox proportional hazards regression was performed to examine the association between age and KS risk. There were 534 incident KS cases with a total follow-up time of 25,134 person-years. The overall KS incidence rate was 2.13 per 100 person-years (95% CI: 1.95–2.32) (Non-HAART users-ever: 5.57 per 100 person-years [95% CI: 5.09–6.10]; HAART users-ever: 0.39 per 100 person-years [95% CI: 0.31–0.51]). Overall, KS frequency and incidence declined with age, even in the oldest age group (*p*_trend_ < 0.0001). However, among non-HAART users-ever, the oldest age group had the highest incidence rate ratio compared to younger individuals [15.01, 95% CI: 6.12–44.22]). While the incidence of KS decreased with age, older HIV-infected persons who do not receive HAART are still at increased risk of KS. As KS remains an important malignancy among HIV-infected persons, earlier HIV diagnoses and HAART initiation, particularly in older HIV-infected persons is warranted.

## Introduction

Kaposi's sarcoma (KS) is caused by human herpesvirus-8 (HHV-8), which was first discovered in 1984 by Chang et al. [1]. There are four different forms of KS: (1) classic KS in elderly men with Jewish ancestry in Mediterranean or Eastern European countries; (2) endemic KS in children with lymphadenopathy in Central and Eastern Africa; (3) iatrogenic KS in immunosuppressed patients after organ transplant; and (4) epidemic KS in HIV-infected populations, which is identified as one of the AIDS-defining malignancies (review in [2]).

AIDS patients have an increased risk of certain types of cancer due to their immunocompromised status [3–6]. As HIV-infected individuals gain more access to highly active antiretroviral therapy (HAART), their life expectancy increases; however, so does their risk of developing cancer [7, 8]. Recent estimates show that there are 1,178,350 people aged 13 and older living with HIV in the United States [9], and more than 24% of people living with AIDS in the United States are 50 years of age and older [10]. By 2015, the cumulative number of AIDS cases over 50 years of age is projected to be 50% of all HIV-seropositive persons in the US [10]. Thus, a growing number of older HIV-infected people are at risk for cancer.

In HIV-infected persons, KS is still one of the most common cancers. KS can have profound effects on quality of life and may become a debilitating condition, especially in the context of the other comorbidities associated with AIDS. An HIV-infected person is 3640 times more likely to have KS than an HIV-uninfected person [11]. Although the incidence of KS has declined tremendously in developed countries owing to the increased access to HAART [3, 12], AIDS-defining malignancies, including KS, remain the most common malignancies among HIV-infected population [13].

Studies of KS among older HIV-infected persons and AIDS patients are limited. Recent reports indicate that cancer incidence has risen rapidly among this aging population [14, 15]. It is possible that KS incidence may increase in spite of HAART as the population ages. Specifically, Biggar et al. [16] reported that the overall risks of all cancers, including KS, among those who are more than 60 years of age was relatively similar to that in young adults with AIDS; however, KS was not separated as a sole outcome of interest in this study. As the HIV-infected population ages, it is important to examine whether the physiological changes associated with age may impact KS risk. We, therefore, sought to examine the age patterns of KS incidence and an association between age and KS risk in an US cohort of HIV-infected men.

## Materials and Methods

### Study population and data collection

Data used for the current study were obtained from the public dataset (release 18) of the Multicenter AIDS Cohort Study (MACS), an ongoing cohort study of HIV-infected and uninfected men who have sex with men (MSM) in the US. MACS is described in detail elsewhere [17]. Briefly, study participants were enrolled in four cities: Baltimore, MD; Chicago, IL; Pittsburgh, PA; and Los Angeles, CA. There were three enrollment periods in MACS: the first enrollment was between April 1984 and April 1985 during which 4954 men were recruited; the second enrollment was between April 1987 and September 1991 during which 668 men were recruited; the third enrollment was between October 2001 and August 2003 during which 1351 men were recruited. The cutoff date for the current analysis was October 1, 2005, covering visits 1 through 43 of MACS.

The MACS study protocol includes a baseline visit and follow-up visits at 6-month intervals. During both baseline and follow-up visits, study participants were administered a standardized questionnaire by trained interviewers. Information obtained through the interview included: sociodemographic and behavioral characteristics (i.e., sexual and other risk factors), health service utilization, medical history, medication use (i.e., HAART and non-HAART drugs), and HIV-related symptoms [17, 18]. Also during baseline and follow-up visits, a standardized medical examination was performed by a physician, physician assistant, or nurse practitioner. Study participants consented to give blood samples for different laboratory tests such as HIV status, lymphocyte phenotypes, Hepatitis B, cytomegalovirus, rapid plasma reagin/fluorescent treponemal antibody, etc. HIV status was tested using enzyme-linked immunosorbent assay and confirmed by Western blot [19]. Standardized flow cytometry was used to quantify T-cell subsets [18, 20] in laboratories participating in the National Institutes of Health/National Institute of Allergy and Infectious Diseases (NIH/NIAID) AIDS Program Flow Cytometry Quality Assessment Program [21, 22]. HIV viral load was measured using RT-PCR (Roche Molecular System, Pleasanton, CA) and performed at the laboratories participating in the NIH/NIAID Virology Quality Assurance Proficiency Testing Program [18].

MACS has recruited 6792 HIV-infected and HIV-uninfected MSM. For the current analyses, participants were excluded if they remained HIV-uninfected for the duration of follow-up (*n* = 3334). Thus, the remaining sample of 3458 men who were either HIV-seropositive at baseline or seroconverted during the cohort was used for the current analysis.

### Variables of interests and measurement

Details on cancer ascertainment and classification were described elsewhere [23]. Briefly, KS ascertainment was performed concurrently during follow-up using different methods such as study interview, medical record abstraction, or vital status review. All cancer site and histology data were documented in MACS using either ICD-O-1 (before 2005) or ICD-O-3 (since December 2005). For standardization, an algorithm was used to convert cancer classifications using ICD-O-1 to ICD-O-3 [23, 24]. For the current analysis, the ICD-O-3 code 9140 (or 9140/3) was used to identify KS cases [25].

Age at time of KS diagnosis/censor was categorized into three groups (<40, 40–49, and ≥50). Race/ethnicity was categorized into three groups (Caucasian American, African American, and other, which included American Indians or Alaskan Native, Asia or Pacific Islanders, and other ethnic groups). Employment status was dichotomized into current employment and no current employment. Education level was grouped into three groups (≤ high school graduate, college level, and graduate level). Individual gross income was categorized into four groups (≤$20,000, $20,000-$39,999, $40,000-$59,999, and ≥$60,000). Current smoking status was defined as “Yes” or “No”.

HAART use was defined as a binary variable (i.e., ever, never), according to the DHHS/Kaiser Panel [26]. Accordingly, a person was defined as being on HAART if he received any combination of two nucleoside reverse transcriptase inhibitor (NRTI) drugs and one of the following: a nonnucleoside reverse transcriptase inhibitor (NNRTI), a protease inhibitor (PI), or an entry inhibitor. Additionally, a person was classified as receiving HAART if any combination of drugs from two of the following classes: NNRTI, PI, integrase inhibitors, and entry inhibitors were identified. Regimens containing the following combinations were not considered HAART: two or more NNRTIs, an NNRTI without a (RTV) boosted PI, and unboosted atazanavir with tenofovir disoproxil fumarate.

### Statistical analysis

Incident cases of KS among HIV-seropositive men were defined as persons who did not have KS at the baseline visit but developed KS during the follow-up period. Incident cases of KS among HIV-seroconverters were defined as persons who did not have KS at the seroconversion visit but developed KS during the follow-up period. The overall time to follow-up was from 1984 (starting at first enrollment) to 2005 (the cutoff period for the provided public dataset). Among HIV-seropositive men, follow-up time was defined for each subject as the time from the date of enrollment into the cohort to the date of diagnosis for KS cases, or to the date of last recorded visit for subjects who did not develop KS. For HIV-seroconverters, follow-up time was from the date of seroconversion to the date of KS diagnosis for KS cases, or from the date of seroconversion to the date of last recorded visit for KS-free participants. Age at diagnosis or last visit was calculated for KS cases or KS-free participants, respectively. It was then, categorized into three groups as mentioned above.

Incidence rates were calculated as the total number of incident KS cases divided by total follow-up time (i.e., person-years) for HIV-seropositive, HIV-seroconverters, and all HIV-infected participants. Poisson distribution was used to estimate 95% confidence intervals (CIs) of incidence rates in each age and race group. Incidence rate ratios (IRRs) and the corresponding 95% CIs were calculated to compare risk between non-HAART users and HAART users across each age and race group. Cox proportional hazards regression was used to calculate hazard ratios (HRs) and respective 95% CIs for KS risk between HAART users and non-HAART users across age and race/ethnicity strata. Since CD4 + cell count and HIV viral load were significantly correlated (*P* < 0.0001), we included only CD4 + cell count into the adjusted models. Covariates included in the models were current employment, education level, individual gross income, current smoking status, CD4 + cell count (as a time-dependent variable), and enrollment period. Enrollment period was included in the adjusted models because a previous analysis using MACS data showed that there was a difference in HAART adherence between Caucasians and African Americans between the two enrollment periods [18]. All statistical analyses were performed using the SAS 9.2 package (Cary, NC) [27]. All tests were two sided and considered significant if the resulting *P*-value was less than 0.05.

## Results

A total of 2884 men were seropositive at baseline, and an additional 574 seroconverted during follow up. Of the men who were seropositive at baseline, 18% were over the age of 50, whereas a higher proportion (30%) of the men who seroconverted over the course of follow up were in the older age group (Table 1). Most study participants were Caucasian. Almost half of study participants were current smokers, in both the HIV-seropositive and seroconverted groups. Also more than 30% of HIV-seropositive persons and 38% of HIV-seroconverters were HAART ever-users (Table 1).

**Table 1:** Baseline sociodemographic characteristics of 3458 HIV-infected men from MACS cohort during 1984–2005.

Characteristics	HIV-seropositive (*n*, %) (*n* = 2884)	HIV-seroconverter (*n*, %) (*n* = 574)
Age		
≤40	1330 (46.1)	182 (31.7)
40–49	1035 (35.9)	219 (38.2)
≥50	519 (18.0)	173 (30.1)
Race/Ethnicity		
Caucasian American	2192 (76.9)	513 (89.4)
African American	455 (16.0)	51 (8. 9)
Others	204 (7.2)	10 (1.7)
Current employment		
No	992 (34.8)	152 (26.5)
Yes	1857 (65.2)	421 (73.5)
Education level		
≤12	652 (23.1)	76 (13.4)
College level	1494 (52.9)	330 (58.1)
Graduate level	678 (24.0)	162 (28.5)
Individual gross income		
≤$20,000	718 (43.3)	111 (28.1)
$20,000–$39,999	460 (27.7)	139 (35.2)
$40,000–$59,999	366 (22.1)	132 (33.4)
≥$60,000	114 (6.9)	13 (3.3)
Current smoking status		
No	1393 (52.0)	306 (54.2)
Yes	1286 (48.0)	259 (45.8)
Packs smoked when smoked most		
Never smoked	124 (6.8)	23 (6.4)
<1/2 pack/day	206 (11.4)	35 (9.8)
1/2 to <1 pack/day	250 (13.8)	42 (11.7)
1 to <2 packs/day	621 (34.3)	116 (32.3)
≥2 packs/day	610 (33.7)	143 (39.8)
HAART use-ever		
No	2001 (69.4)	355 (61.8)
Yes	883 (30.6)	219 (38.2)
Enrollment		
1	1814 (62.9)	514 (89.6)
2	382 (13.2)	26 (4.5)
3	688 (23.9)	34 (5.9)
CD4 + cell count (mean ± SD)	359 ± 272	523 ± 344
HIV viral load (mean ± SD)	49,945 ± 102,423	151,978 ± 675,846

MACS, Multicenter AIDS Cohort Study; SD, standard deviation.

During the 1984–2005 period included in the current analysis, we identified 534 KS cases with total follow-up time of 25,134 person-years. Therefore, the overall KS incidence rate was 2.13 per 100 person-years (95% CI: 1.95–2.32). The KS incidence rate among HIV-seropositives was 2.28 per 100 person-years (95% CI: 2.08–2.50) and among HIV-seroconverters was 1.41 per 100 person-years (95% CI: 1.10–1.81). The incidence of KS was 1.85 (95% CI: 1.60–2.14) per 100 person-years in the 40–49 age group, while the incidence was much lower among the ≥50 age group (0.39 per 100 person-years [95% CI: 0.27–0.55] Table 2). Caucasians had the highest KS incidence rate (2.35 per 100 person-years, 95% CI: 2.14–2.57) while African Americans had the lowest (0.85 per 100 person-years, 95% CI: 0.54–1.27) (Table 2).

**Table 2:** Incidence of Kaposi's sarcoma by HIV-serostatus among 3458 men from MACS cohort during 1984–2005.

	Total HIV-infected (*n* = 3458)			HIV-seropositive only (*n* = 2884)			HIV-seroconverter only (*n* = 574)		
	Incident case	Follow-up time (years)	Incidence rate (95% CI)	Incident case	Follow-up time (years)	Incidence rate (95% CI)	Incident case	Follow-up time (years)	Incidence rate (95% CI)
Age									
<40	308	5906	5.21 (4.66–5.84)	271	5245	5.17 (4.60–5.84)	37	661	5.60 (3.86–7.72)
40–49	191	10,315	1.85 (1.60–2.14)	171	8450	2.02 (1.73–2.37)	20	1865	1.07 (0.65–1.65)
≥50	35	8913	0.39 (0.27–0.55)	29	6969	0.42 (0.28–0.60)	6	1944	0.31 (0.11–0.67)
			*P*_trend_ <0.0001			*P*_trend_ <0.0001			*P*_trend_ <0.0001
Race/Ethnicity									
Caucasian	497	21,132	2.35 (2.14–2.57)	438	16,983	2.58 (2.34–2.84)	59	4149	1.42 (1.09–1.83)
African American	23	2711	0.85 (0.54–1.27)	20	2422	0.83 (0.50–1.27)	3	289	1.04 (0.21–3.03)
Others	14	1161	1.21 (0.66–2.03)	13	1129	1.15 (0.61–1.97)	1	32	3.12 (0.08–17.4)

CI, confidence interval; MACS, Multicenter AIDS Cohort Study.

Table 3 shows the incidence of KS in HAART users and non-HAART users across age and race/ethnicity groups. The KS incidence in both HAART and non-HAART users declined with age (*P*_trend_ < 0.0001). Those who did not receive HAART were significantly more likely to develop KS than those who did receive HAART in each of the age groups <40, 40–49, and ≥50 (IRR = 2.25 [95% CI: 1.50–3.51]; 7.49 [95% CI: 4.96–11.71]; and 15.01 [95% CI: 6.12–44.22]; respectively, *P*_trend_ < 0.0001). The protective effect of HAART was also observed in all three race/ethnicity groups. A similar observation was found when stratifying by HIV-seropositive only versus HIV-seroconverters only (Table 3).

**Table 3:** Incidence of Kaposi's sarcoma by HAART use status among 3458 men from MACS cohort during 1984–2005.

	HAART Users-Ever (*n* = 1102)			Non-HAART Users-Ever (*n* = 2356)			IRR (95% CI)^2^
		
	Incident case	Follow-up time (years)^1^	Incidence rate (95% CI)	Incident case	Follow-up time (years)^1^	Incidence rate (95% CI)	
Total HIV-Infected							
Age							
<40	26	1015	2.56 (1.67–3.77)	282	4891	5.77 (5.11–6.51)	2.25 (1.50–3.51)
40–49	27	5696	0.47 (0.3–0.69)	164	4619	3.55 (3.03–4.16)	7.49 (4.96–11.71)
≥50	6	6742	0.09 (0.03–0.19)	29	2171	1.26 (0.90–1.92)	15.01 (6.12–44.22)
*P*_trend_			<0.0001			<0.0001	<0.0001
Race/Ethnicity							
Caucasian	53	11,457	0.46 (0.35–0.61)	444	9675	4.59 (4.18–5.07)	9.92 (7.44–13.45)
African American	4	1316	0.30 (0.08–0.78)	19	1395	1.36 (0.82–2.12)	4.48 (1.49–18.11)
Others	2	580	0.34 (0.04–1.24)	12	581	2.06 (1.07–3.61)	5.99 (1.33–55.10)
HIV-seropositive only							
Age							
<40	16	891	1.80 (1.03–2.91)	255	4354	5.86 (5.17–6.64)	3.26 (1.97–5.79)
40–49	21	4546	0.46 (0.29–0.71)	150	3904	3.84 (3.26–4.52)	8.32 (5.25–13.83)
≥50	4	5212	0.08 (0.02–0.20)	25	1757	1.42 (0.92–2.10)	18.54 (6.40–73.30)
*P*_trend_			<0.0001			<0.0001	<0.0001
Race/Ethnicity							
Caucasian	37	8816	0.42 (0.30–0.57)	401	8167	4.91 (4.45–5.42)	12.37 (8.75–18.01)
African American	2	1167	0.17 (0.02–0.62)	18	1255	1.43 (0.85–2.66)	8.37 (2.00–74.37)
Others	2	566	0.35 (0.04–1.27)	11	563	1.95 (0.97–3.50)	5.53 (1.20–51.34)
HIV-seroconverter only							
Age							
<40	10	124	8.06 (3.87–14.84)	27	537	5.03 (3.31–7.34)	0.62 (0.29–1.44)
40–49	6	1150	0.52 (0.19–1.14)	14	715	1.96 (1.07–3.29)	3.75 (1.35–11.92)
≥50	2	1530	0.13 (0.02–0.47)	4	414	0.97 (0.26–2.47)	7.39 (1.06–81.71)
*P*_trend_			<0.0001			<0.0001	<0.0001
Race/Ethnicity							
Caucasian	16	2641	0.61 (0.35–0.98)	43	1508	2.85 (2.06–3.85)	4.71 (2.60–8.95)
African American	2	149	1.34 (0.16–4.85)	1	140	0.71 (0.02–3.98)	0.53 (0.01–10.22)
Others	0	14	–3	1	18	5.55 (0.14–30.9)	–3

CI, confidence interval; IRR, incidence rate ratio.

1 Follow-up time for both KS and non-KS incidence cases;

2 Comparison between non-HAART users versus HAART users using poisson distribution.

3 Small number, estimates could not be calculated.

Table 4 presents the results of a multivariable model that determined the association between age and race with KS risk in non-HAART and HAART users. The risk of KS decreases with age in both groups; however, the hazard ratio in the oldest age group is higher than those aged 40–49 (Non-HAART users: HR = 0.26 [95% CI: 0.14–0.45]; and 0.63 [95% CI: 0.45–0.87] in age group 40–49 and ≥50, respectively; HAART users: HR = 0.01 [95% CI: 0.002–0.02]; and 0.07 [95% CI: 0.04–0.15] in age group 40–49 and ≥50, respectively). Between race/ethnic groups, the risk of KS was not significantly different regardless of HAART status. Due to small numbers of subjects, we could not calculate the hazard ratio for the other race/ethnicity group in the adjusted model.

**Table 4:** Adjusted risks of KS within each group of non-HAART and HAART users among 3458 men from MACS cohort during 1984–2005 (Cox proportional hazards regression model).

	KS incidence cases	Follow-up time (person-years)^3^	Un-adjusted Model HR (95% CI)	Adjusted model HR (95% CI)
Non-HAART Users-ever^1^				
Age				
<40	282	4891	Ref.	Ref.
40–49	164	4619	0.21 (0.14–0.32)	0.26 (0.14–0.45)
≥50	29	2171	0.54 (0.44–0.65)	0.63 (0.45–0.87)
*P*_trend_			<0.0001	
Race/Ethnicity				
Caucasian	444	9675	Ref.	Ref.
African American	19	1395	0.34 (0.21–0.53)	0.54 (0.25–1.18)
Others	12	581	0.51 (0.29–0.91)	0.86 (0.35–2.09)
HAART Users-ever^2^				
Age				
<40	26	1015	Ref.	Ref.
40–49	27	5696	0.02 (0.01–0.06)	0.01 (0.002–0.02)
≥50	6	6742	0.12 (0.07–0.22)	0.07 (0.04–0.15)
*P*_trend_			<0.0001	
Race/Ethnicity				
Caucasian	53	11,457	Ref.	Ref.
African American	4	1316	0.73 (0.27–2.04)	0.28 (0.04–2.58)
Others	2	580	0.87 (0.21–3.60)	–4

CI, confidence interval; KS, Kaposi's sarcoma; HR, hazard ratio.

1 Adjusted model included: age, race/ethnicity, Individual gross income, education level, employment status, Enrollment.

2 Adjusted model included: age, race/ethnicity, individual gross income, education level, employment status, enrollment, CD4 + cell count.

3 Follow-up time for both KS and non-KS incidence cases.

4 Small number, estimates could not be calculated.

## Discussion

In the current analysis, we examined the age patterns of KS incidence among HIV-infected MSM, using the public dataset from MACS, an ongoing prospective study in the US. We found that KS incidence in HIV-infected men declined with age. We, however, observed that compared to those treated with HAART, the risk of KS among HIV-infected men not treated with HAART was increased, and the risk was the highest among men more than 50 years old.

The overall incidence of KS in our analysis (2.13 per 100 person-years [95% CI: 1.95–2.32]) is similar to that found in the HIV/AIDS Cancer Match Study (HACM) that examined Caucasian MSM from six states and five metropolitan areas during the 1981–1996 period (incidence rate: 2.4 per 100 person-years) [16]. Given that more than 80% of our sample is Caucasian, we believe that our study population is comparable to theirs. Our finding is also consistent with recent estimates from HACM using data from all 50 states in which they found that the KS incidence rate was 2.5 per 100 person-years [3]. In 2010, an estimate of KS incidence from the MACS study showed that the race- and age-standardized KS incidence ratio (SIR) was 139.10 (compared with US general population-SEER) [23]. However, this analysis did not present the incidences IRRs for specific age groups.

The declining trend of KS incidence with age found in our analysis is also consistent with findings from other studies [16, 28, 29]. The trend of KS incidence peaking around ages 30–39 and then declining in the later age groups has also been reported in previous studies of younger MSM with AIDS [28, 29] and from the HACM study [3, 16]. There can be multiple reasons behind this trend. In this study, the numbers of people in the older age groups are smaller than in the younger age groups; thus, the incidence estimates from these sparser categories may be less reliable. This problem may also be compounded by the fact that in the current analysis, more than 46% of study participants from enrollments 1 and 2 died at some point during the follow-up period, thus fewer participants from those cohorts survived to age 50. Additionally, the introduction of HAART had a great impact on the declining trend of KS incidence, which has been reported by previous studies [12, 30–34], further decreasing the number of cases.

Another important point from our analysis is that although there is a declining trend of KS incidence with age, the risk of KS development is higher in every age group among those without HAART compared to those with HAART (*P*_trend_ < 0.0001). Also, the IRR of KS was the highest in the oldest age groups for both HIV-infected men and HIV-seroconverters. Although the incidence of KS is decreasing, it is important to note that KS and non-Hodgkin lymphoma are still the most common cancers in the US AIDS population [3] despite the availability of HAART. In fact, a recent estimate showed that 45% of eligible HIV/AIDS patients in the United States are not on HAART [35]. Furthermore, one study using data from MACS and the Tri-Service AIDS Clinical Consortium reported that there may be a delayed effect of HAART on AIDS-defining cancers during the HAART period [31]. Specifically, the relative risk of AIDS-defining cancers (including KS and non-Hodgkin lymphoma) with a 1-year lag period (1996–1998) was 0.23 [95% CI: 0.12–0.42], 2-year lag period (1999–2002) was 0.24 (95% CI: 0.13–0.45) and 3-year lag period (2003–2006) was 0.29 (95% CI: 0.15–0.55). Additionally, a recent study in 965 Egyptians by Mbulaiteye et al. [36] reported that participants aged more than 45 are 5.3 times (95% CI: 2.2–13.2) more likely to have HHV-8 seropositivity than younger participants (15–24 years of age). Taken together, these data suggested that the risk of KS development among older HIV-infected persons among those who are not on HAART appears to be increased, and without a comprehensive prevention, care and treatment program focused on early identification and treatment of this population, KS will continue to cause significant morbidity and mortality among older HIV-infected individuals. Additionally, there are two studies by Oursler et al. [37, 38] reporting that treatment might not be as effective or may have more adverse effects in elderly patients. They also reported that while on HAART, older patients had a reduced ability to exercise and lower functional performance, two important indicators of “frailty syndrome.” Because HAART adherence and treatment is different between older HIV-infected persons and younger HIV-infected persons [39–41], particular attention (i.e., dose and length of HAART as well as their interaction with other drugs) is warranted from clinicians who treat the older HIV-infected population.

We also found that the incidence of KS decreased in both older age groups; however, in multivariable analyses, the risk of KS in the oldest group was higher than that in 40- to 49-year-olds. Further studies are, therefore, warranted to confirm the KS incidence patterns among the older population with or without HAART usage.

The main strength of our analysis was the use of the largest cohort study among HIV-infected men in the US to examine the age patterns of KS incidence. This well-designed cohort allowed us to calculate accurate estimates of KS incidence with age. The design of this cohort also makes it possible to identify the date of seroconversion and to calculate follow-up time accurately. Also, too our knowledge, current analysis is one of the only studies to examine KS in HIV-seroconverters. One limitation of this analysis is that because the cohort only included MSM and most of them were Caucasian American, our results may not be generalizable to HIV-infected women or to mixed race/ethnic populations. Another limitation is that since the data provided were followed to 2005, the definition of HAART used in our study might not be comparable to the new definition in 2011, particularly because several new HIV medications have been approved since 2005. The other limitation is that information on KSHV serostatus was not available in the current provided dataset.

In summary, we found declining incidence rates of KS with age among HIV-infected men in the largest US cohort. Furthermore, we reported that HAART has great impact in decreasing the overall KS incidence in this cohort. Among the HIV-infected older population, the KS risk is higher in those without HAART compared to those with HAART. Our findings are consistent with results from previous studies and provide information on the directions of age and period effects on KS risk. Our results have substantial clinical and public health implications related to the low usage of HAART in HIV/AIDS-affected populations, especially in older populations. As KS remains an important malignancy among HIV-infected persons, earlier HIV diagnosis and HAART initiation, particularly in older HIV-infected persons should remain a public health priority.
